# Universal primers for rift valley fever virus whole-genome sequencing

**DOI:** 10.1038/s41598-023-45848-z

**Published:** 2023-10-31

**Authors:** Kwan Woo Kim, Banseok Lee, Sujeong Eom, Donghoon Shin, Changwoo Park, Seil Kim, Hana Yi

**Affiliations:** 1 Department of Public Health Sciences, Graduate School, Korea University, Seoul, Republic of Korea; 2 Interdisciplinary Program in Precision Public Health, Korea University, Seoul, Republic of Korea; 3 Integrated Biomedical and Life Science, Graduate School, Korea University, Seoul, Republic of Korea; 4 Microbiological Analysis Team, Group for Biometrology, Korea Research Institute of Standards and Science (KRISS), Daejeon, Republic of Korea; 5 Convergent Research Center for Emerging Virus Infection, Korea Research Institute of Chemical Technology (KRICT), Daejeon, Republic of Korea; 6 Department of Agricultural Biotechnology, Seoul National University, Seoul, Republic of Korea; 7 Department of Bio-Analysis Science, University of Science and Technology, Daejeon, Republic of Korea; 8 School of Biosystems and Biomedical Sciences, Korea University, Seoul, Republic of Korea; 9 Present Address: Center for Study of Emerging and Re-emerging Viruses, Korea Virus Research Institute, Institute for Basic Science (IBS), Daejeon, Republic of Korea

**Keywords:** Genomics, Genetic variation, DNA sequencing, Next-generation sequencing, Viral genetics

## Abstract

Rift Valley fever (RVF) is a mosquito-borne zoonotic disease causing acute hemorrhagic fever. Accurate identification of mutations and phylogenetic characterization of RVF virus (RVFV) require whole-genome analysis. Universal primers to amplify the entire RVFV genome from clinical samples with low copy numbers are currently unavailable. Thus, we aimed to develop universal primers applicable for all known RVFV strains. Based on the genome sequences available from public databases, we designed eight pairs of universal PCR primers covering the entire RVFV genome. To evaluate primer universality, four RVFV strains (ZH548, Kenya 56 (IB8), BIME-01, and Lunyo), encompassing viral phylogenetic diversity, were chosen. The nucleic acids of the test strains were chemically synthesized or extracted via cell culture. These RNAs were evaluated using the PCR primers, resulting in successful amplification with expected sizes (0.8–1.7 kb). Sequencing confirmed that the products covered the entire genome of the RVFV strains tested. Primer specificity was confirmed via in silico comparison against all non-redundant nucleotide sequences using the BLASTn alignment tool in the NCBI database. To assess the clinical applicability of the primers, mock clinical specimens containing human and RVFV RNAs were prepared. The entire RVFV genome was successfully amplified and sequenced at a viral concentration of 10^8^ copies/mL. Given the universality, specificity, and clinical applicability of the primers, we anticipate that the RVFV universal primer pairs and the developed method will aid in RVFV phylogenomics and mutation detection.

## Introduction

Rift Valley fever (RVF) is a zoonotic disease affecting both animals and humans. The causal pathogen, a mosquito-borne virus, was first reported in young sheep and humans in the Rift Valley of Kenya in 1931^1^. Although initially confined to sub-Saharan Africa, the disease has been reported in other regions beyond the African continent since 2000, including Saudi Arabia, Yemen, and China^2,3^. Typically, dozens to hundreds of cases of RVF infection occur every 2–3 years. In cases where livestock are affected by this disease, a characteristic symptom known as the “abortion storm” leads to high mortality rates and miscarriage rates. The spread of RVF causes significant economic damage to livestock production and rural farming communities^4,5^.

The incubation period of the disease is approximately 2–6 days, and mortality rates in humans are estimated to be 0.5–2.0%^6^. The majority of patients exhibit mild symptoms, including fever, dizziness, and severe weight loss, and naturally recover within 2–7 days. However, symptoms such as shock or encephalitis can occur in severe cases. The main biological vectors of RVF, through which it is transmitted to both livestock and humans, include more than 30 mosquito species belonging to the genera *Aedes*, *Culex*, *Anopheles*, *Eretmapodites*, *Mansonia*, and *Coquillettidia*^7^. Despite the serious nature of RVF infection in both humans and livestock, there are currently no effective vaccines or treatments available for this disease. Veterinary vaccines and attenuated vaccines have been developed, but vaccines for human use have not been developed yet^4^. Hence, in 2018, RVF was designated as a “Disease X” priority disease by the World Health Organization (WHO)^8^.

The RVF virus (RVFV) genome comprises three single-stranded RNA segments designated as large, medium, and small segment. The large segment (L; 6.4 kb) encodes an RNA-directed RNA polymerase^9^. The medium segment (M; 3.8 kb) encodes two envelope glycoproteins (Gn and Gc) and the NSm protein^10–12^. The small segment (S; 1.6 kb), which is characterized by ambisense polarity, encodes a structural nucleoprotein (N) in the negative sense and a small nonstructural protein (NSc) in the positive sense^13^.

Since its initial discovery, RVFV has spread across a wide geographical range, and, to date, only a single serotype has been identified^14^. Approximately 184 full-length genomes of RVFV have been deposited in the Virus Pathogen Resource (ViPR) database; they have low genetic diversity and differ by less than 5% and 2% at the nucleotide (nt) and amino acid (aa) levels (M segment: nt, 5%; aa, 2%; and L and S segments: nt, 4%; aa, 1%), respectively. However, phylogenetic tree analysis based on the whole genome has revealed several distinct genetic lineages (A to O) of RVFV^15^. This diversity is believed to be attributable to the reassortment of the segmented genome^16,17^, and despite high genome sequence similarity, RVFV is phylogenetically branched into 15 lineages^15^.

RVFV diagnostic methods include quantitative real-time PCR (qRT-PCR)^18^, multiplex PCR-based microarrays^19^, and recombinase polymerase amplification (RPA)^20^. The genes *NSm*, *NSs*, and *Gc* are used for qRT-PCR^18,21,22^. Although these methods are considerably valuable for the rapid and accurate diagnosis of viral infections, the methods are of limited utility with respect to characterizing specific mutations in a genome-wide manner. Viral mutations are of concern because they can influence the transmissibility of the virus, severity of infection, and the efficacy of vaccines. This highlights the need for the rapid identification of mutations, as exemplified by the mutations in the SARS-CoV-2 virus, which has had considerable effects on virus transmission and vaccine efficacy^23–25^. Consequently, the WHO recommends whole-genome analysis for the accurate diagnosis of infectious viruses and the identification of associated mutations. Sequencing of viral genomes requires the initial isolation of viral agents from clinical samples preserved in cell culture, which takes several days. To obtain genome information immediately, it is necessary to extract and sequence viral nucleic acids directly from clinical samples. However, the amount of viral nucleic acids in clinical samples is generally insufficient for full-genome analysis; hence, initial sample amplification is required. For example, the ARTIC nCoV-2019 Amplicon Panel has been used for the whole-genome analysis of SARS-CoV-2^26,27^. MERS-CoV and the alphacoronavirus universal primer panel has been developed for genome surveillance^28,29^. At present, however, no universal primers are available for the amplification of RVFV nucleic acid.

The RVFV genome does not show specific regions where mutations are known to frequently occur. The mutations found in the RVFV genome are a result of random single-nucleotide polymorphisms (SNPs) rather than from specific segments. As mentioned earlier, due to the lack of serotype differentiation in RVFV, complete genomic information is crucial for distinguishing different strains^14,30^. Therefore, the development of universal primers for RVFV could serve as a useful tool for monitoring RVFV mutations and determining the genetic diversity of the virus. Currently, the RVFV genome is being characterized by the following methods: Genome sequences are determined via sequence-independent single primer amplification (SISPA) and primer walking with Sanger sequencing^31,32^. Target sequences of the nonstructural (*Ns*) gene and glycoprotein (*Gn*) gene are used for phylogenetic analyses^33^.

The aim of this study was to develop universal RVFV primers that could be applied to RVFV amplicon-based whole-genome sequencing. To verify the applicability of the developed primers, we assessed the nucleic acids of four representative viral strains from different phylogenetic branches of RVFV. Additionally, mock clinical samples were prepared to evaluate the applicability of the developed methodology to the analysis of patient clinical samples.

## Results

### Selection of universal primers for RVFV

**Figure 1:**
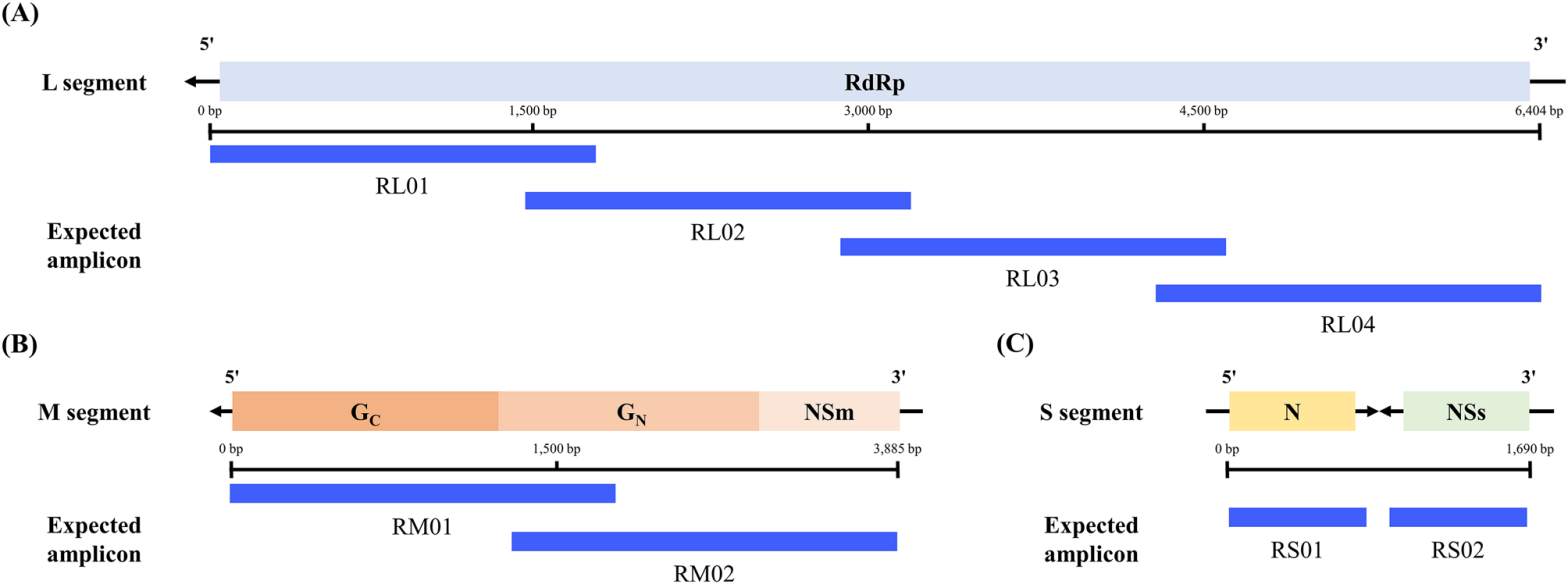
Position of the designed universal primers and the expected amplicons alongside the genome of RVFV. The graphical image of the (**A**) L segment, (**B**) M segment, and (**C**) S segment of the RVFV genome shows the length and gene contents of the RNA genome. The localization of expected PCR amplicons from the eight universal primers designed in this study are indicated as blue solid lines.

**Figure 2:**
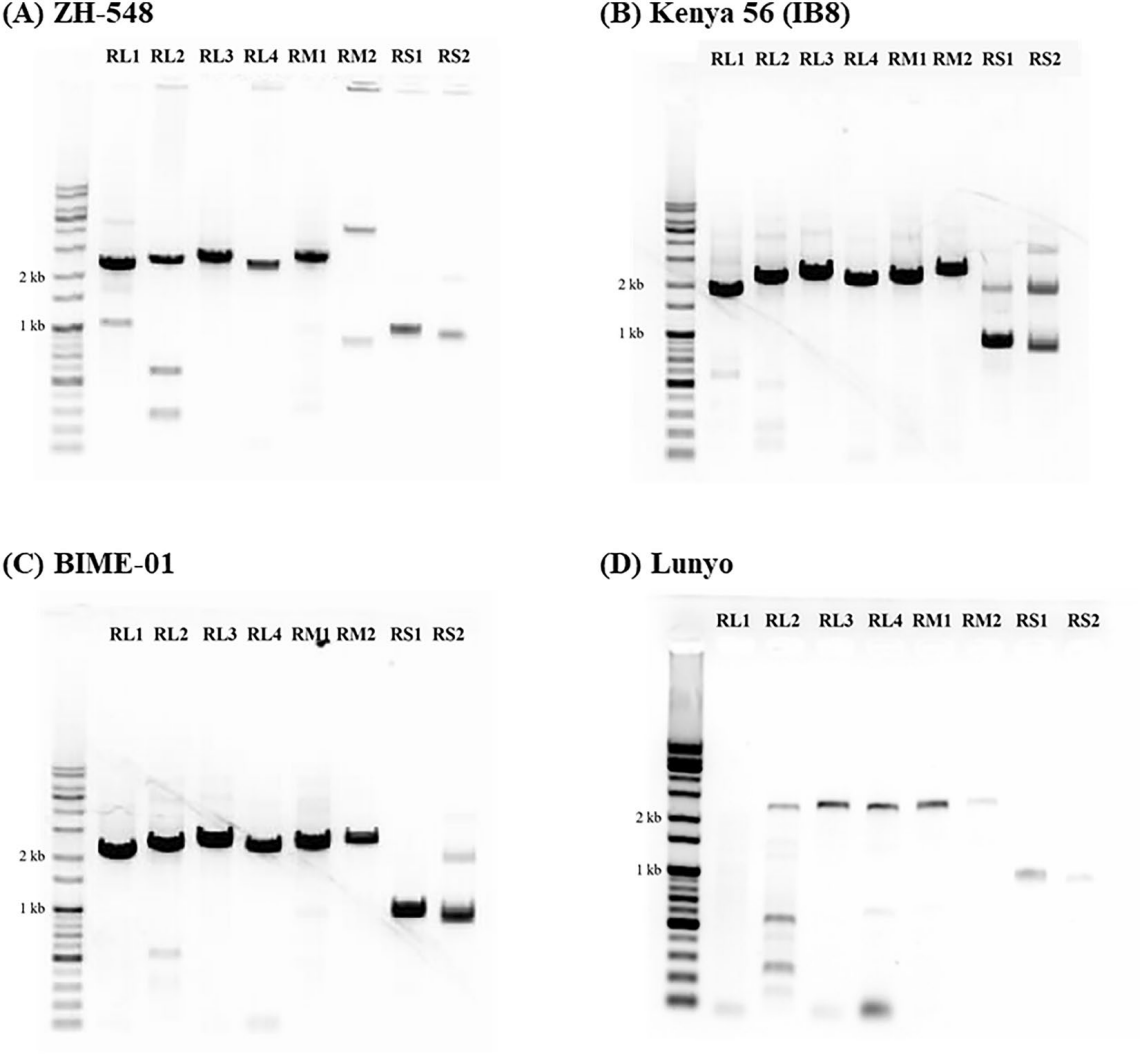
Experimental amplification products of RVFV universal primers. Agarose gel electrophoresis image of PCR amplicons from the four test strains of RVFV. (**A**) ZH548, (**B**) Kenya 56 (IB8), (**C**) BIME-01, and (**D**) Lunyo.

A total of 80 candidate universal primers were designed based on the publicly available genomes of RVFV strains. To select the most optimal universal primers, candidate primers were screened via PCR analysis using the four RVFV strains (ZH548, Kenya 56 (IB8), BIME-01, and Lunyo). Eight universal primer pairs for RVFV covering the entire RVFV genome segments and physical regions were selected (Table 1, Fig. 1). Gel electrophoresis analysis of the PCR amplicons revealed products of the expected size (0.8–1.7 kb) (Fig. 2). However, several unexpected phenomena were observed, listed as follows. (1) The M2 amplicon of the ZH548 strain appeared different from its theoretically expected size. However, Sanger sequencing of this specific band revealed that the target sequence was amplified with the correct size and sequence. (2) In all the four strains tested, we observed the generation of multiple bands in L2 and S2 amplicons. However, we decided to retain those primers due to the absence of alternative primer candidates for those regions. (3) The amplification efficiency of L1 decreased according to the decrease in viral RNA concentration. This was problematic because the lower amplification efficiency than that of other primer regions could result in the failure of sequencing of corresponding regions. Because the search for better primers for this region did not succeed, four additional primer pairs were designed and used together in the further analyses of this problematic region. (4) Evaluation of the potential applicability of multiplex tiling PCR revealed a lack of S-segment synthesis in the amplicon of the Lunyo strain (Supplementary Fig. 1), indicating that the selected primer pairs would not be suitable for multiplex tiling PCR.

**Table 1:** List of RVFV universal primers for whole genome sequencing.

Target region	Primers	Sequence (5' → 3')	Product size (bp)
RL01	RL01-1F	ACACAAAGGCGCCCAATCA	1,737
	RL01-1737R	AAACCCTGGAAGTGGAGAG	
RL02	RL02-1472F	CATCAGCCATCCTTRTCAG	1,957
	RL02-3428R	GCTGGGAAGCTGATTAGCA	
RL03	RL03-2962F	CCTGTGTGGACTTGTGC	2,066
	RL03-5027R	GATTCCTTTATCTCAACGTTTG	
RL04	RL04-4471F	TCACTGGATTCTATAGTTTAC	1,914
	RL04-6384R	ACACAAAGACCGCCCAATATT	
RM01	RM01-2F	ACACAAAGACGGTGCATTAAAT	2,036
	RM01-2037R	AGGTATGTGCTATAACGTGG	
RM02	RM02-1650F	GATGATCAGTCAGTTAGCTC	2,196
	RM02-3845R	TCAAAGAGTTAGTTTAATTCCY	
RS01	RS01-1F	ACACAAAGACCCCCTAGTG	818
	RS01-818R	AACCTCTARTCAACCTCAAC	
RS02	RS02-907F	TGGGCAGCCACTTAGGCT	764
	RS02-1671R	ACACAAAGCTCCCTAGAGAT

### Genome sequences determined from primer amplicons

For each of the four RVFV strains (ZH548, Kenya 56 (IB8), BIME-01, and Lunyo), purified amplification products were pooled at the same concentration and sequenced using the Oxford Nanopore Technologies (ONT; Oxford, United Kingdom) MinION Mk1C sequencer and the Illumina (San Diego, CA, USA) MiSeq platform.

The sequencing statistics of MinION were as follows: (1) Strain ZH-548 produced 3.2 Gb (fast5 20 GB, 1,252,527 reads) of raw data and four linear contigs (17,132.8–237.223.1X sequencing depth) after assembly. (2) Kenya 56 (IB8) produced 574.34 Mb (fast5 3.36 GB, 198,768 reads) of raw data and four linear contigs (15,376–21,778.55X sequencing depth). (3) BIME-01 produced 4.22 Gb (fast5 26.1 GB, 1,434,586 reads) of raw data and four linear contigs (108,352.3–367,227.3X sequencing depth). (4) Lunyo produced 3.55 Gb (fast5 25.3 GB, 2,147,677 reads) of raw data and four linear contigs (43,571.9–280,659.9X sequencing depth). A mean Phred score of 13 (quality filtering > 8) and read lengths greater than 800–1600 bp (length filtering > 700 bp) were observed.

The sequencing statistics of MiSeq were as follows: (1) Strain ZH-548 produced 2.18 Gb (3,466,421 reads) of raw data and four linear contigs (99,154.9–285,104.7X sequencing depth) after assembly. (2) Kenya 56 (IB8) produced 2.57 Gb (4,089,726 reads) of raw data and four linear contigs (116,523.2–564,624.9X sequencing depth). (3) BIME-01 produced 2.15 Gb (3,420,329 reads) of raw data and four linear contigs (94,967.6–351,259.8X sequencing depth). (4) Lunyo produced 3.16 Gb (5,025,765 reads) of raw data and four linear contigs (83,987.5–762,540.4X sequencing depth). A mean Phred score of 30 was observed.

The MinION sequences of all four strains were successfully assembled via reference mapping. The mean sequencing read depth and coverage of MinION were 15,376–367,227X and 99.54–100%, respectively (Table 2). The MiSeq sequences of all four strains were successfully assembled based on reference mapping. Compared to the mapping method, the M segment of strain ZH548 was not assembled during the de novo assembly and showed lower genome coverage, thereby indicating that the reference mapping method is more suitable for RVFV genome assembly (Supplementary Table 1). The mean sequencing read depth and coverage of MiSeq were 28,189–475,170X and 99.54–100%, respectively (Table 2).

**Table 2:** Comparison of whole-genome sequencing data according to viral strains and sequencing platforms.

Strains	Platform	% Mapped read* (L/M/S1/S2)	% Genome coverage** (L/M/S1/S2)	% Similarity***
ZH-548	MinION	30.58/30.78/23.86/17.67	100/99.5367/100/100	100
	MiSeq	47.67/24.57/13.23/14.35	100/99.5367/100/100	100
Kenya 56 (IB8)	MinION	34.46/20.73/14.08/39.94	100/99.5367/100/100	100
	MiSeq	44.55/22.00/10.69/23.36	100/99.5367/100/100	100
BIME-01	MinION	37.18/17.33/22.67/29.40	100/99.5367/100/100	100
	MiSeq	48.46/22.06/16.00/14.29	100/99.5367/100/100	100
Lunyo	MinION	18.50/6.50/4.92/17.76	100/99.5367/100/100	100/99.9482/100/100
	MiSeq	20.69/14.47/5.86/2.130	100/99.5367/100/100	100

*The mapped read refers to the proportion of read counts that align to the reference sequence for each segment among the raw reads generated from the NGS sequencing.

**Genome coverage means the percentage area coverage of a reference (consensus sequence; L 6,404 bp; M, 3,867 bp; S1, 738 bp; S2, 798 bp) by the assembled contig.

***Similarity means the proportion of matched nucleotides in the assembled contigs compared to a reference (consensus sequence; L, 6,404 bp; M, 3,867 bp; S1, 738 bp; S2, 798 bp).

In both MinION and MiSeq sequencing, the determined genome sequences showed 100% sequence similarity to the reference sequence with only one exception. The Lunyo M segment showed 99.9482% of similarity to the known sequence of reference genome (Table 2). The newly determined sequences of the four test strains are deposited in GenBank (accession number: OQ440142-OQ440157).

**Figure 3:**
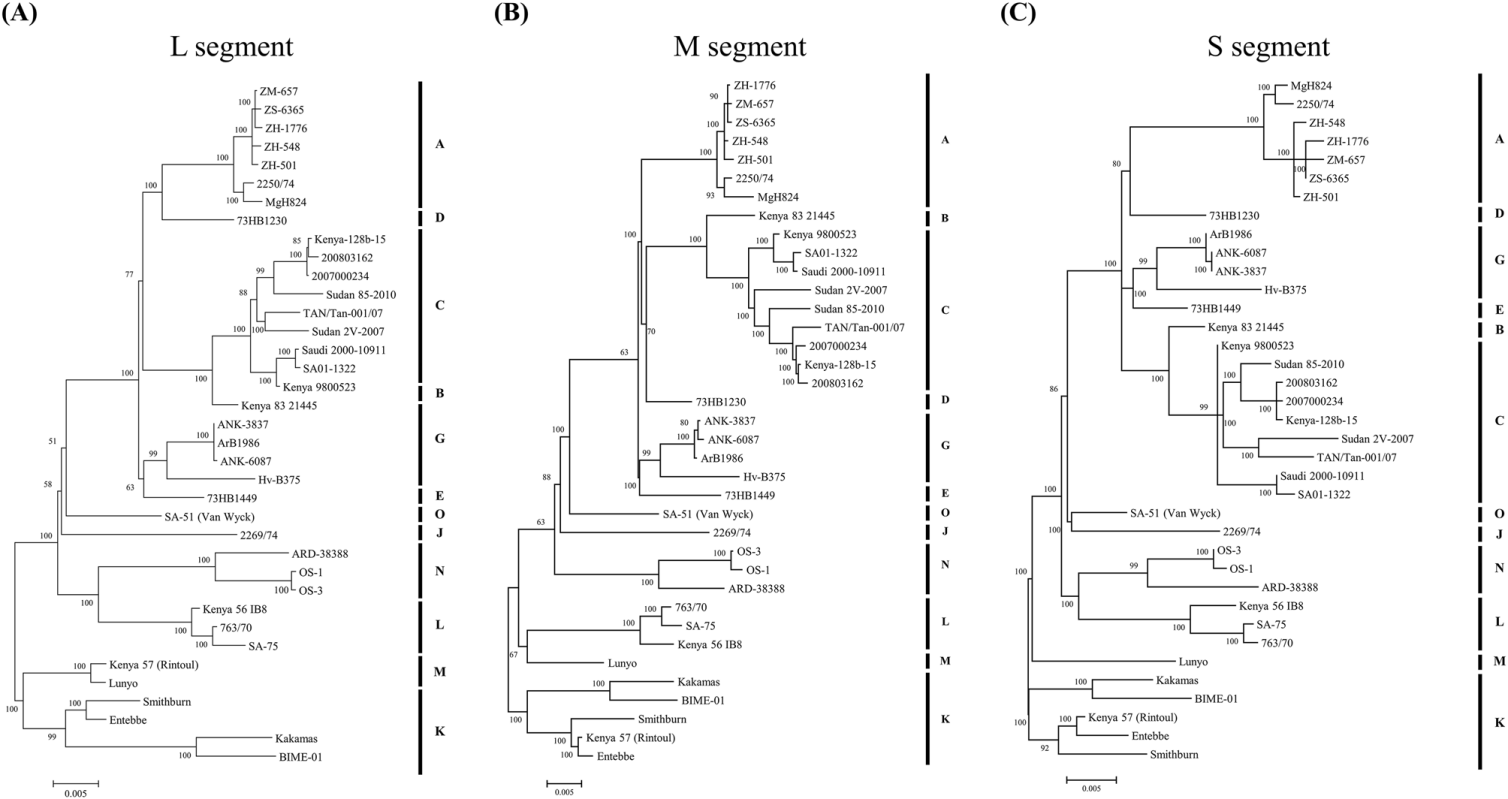
Phylogenetic tree based on the whole-genome sequences of RVFV. Maximum likelihood tree of the (**A**) L segment, (**B**) M segment, and (**C**) S segment. Tree analysis was performed using RAxML version 8.2.10 and evaluated using 100,000 bootstrap replicates. Lineage names followed the nomenclature of Grobbelaar et al.^11^.

Phylogenetic analysis was performed using the newly determined genome sequences. Based on the partial 490-nt M segment method proposed by Grobbelaar et al.^15^, which classifies strains into 15 lineages from A to O, the ZH-548, Kenya 56 (IB8), BIME-01, and Lunyo strains are members of lineages A, L, K, and M, respectively (Fig. 3, Supplementary Fig. 2), in line with previous reports.

### Evaluation of primer specificity, versatility, and efficiency

The performance of universal primer pairs was further evaluated in terms of specificity, versatility, amplification efficiency, and sensitivity. The specificity of the selected universal primer pairs for RVFV was assessed by in silico comparison against known nucleotide sequences. A BLASTn search was conducted on the NCBI nr/nt database to determine if the selected universal primers shared similarities with other genomes, including those of the sandfly fever Naples phlebovirus and the human genome. No similarities (E-value < 10^−5^) were observed with any genome besides RVFV, demonstrating the high specificity of the universal primer set for RVFVs.

To evaluate the versatility of the universal primer pairs, each primer pair was aligned to the 37 genomes of lineages A to O of RVFV. There was a 0 or 1 mismatch between individual primer pairs and genome sequences, and 20-nt mismatches were confirmed for all eight primers (Supplementary Fig. 3), with the exception of the SA 51 (Van Wyck) (DQ380195) strain, for which two mismatches were obtained using the M02 reverse primer, and the TAN/Tan 001/07 (HM586970) strain, which showed three mismatches with the S01 forward primer. The sequences of the regions with mismatches in the SA 51 (Van Wyck) and TAN/Tan 001/07 strains differed from those of closely related strains, as indicated by the phylogenetic analysis. In addition, the content of the mismatch was observed in the middle of homopolymeric sequences (with the same base sequence continuously in the problematic region). Hence, the 2–3 mismatches observed here were considered sequencing errors occurring at the ends of the sequences (Supplementary Table 2). Consequently, assuming that these mismatches are attributable to sequencing errors, it would appear that the primer pairs developed in this study are universal for all RVFV lineages.

**Figure 4:**
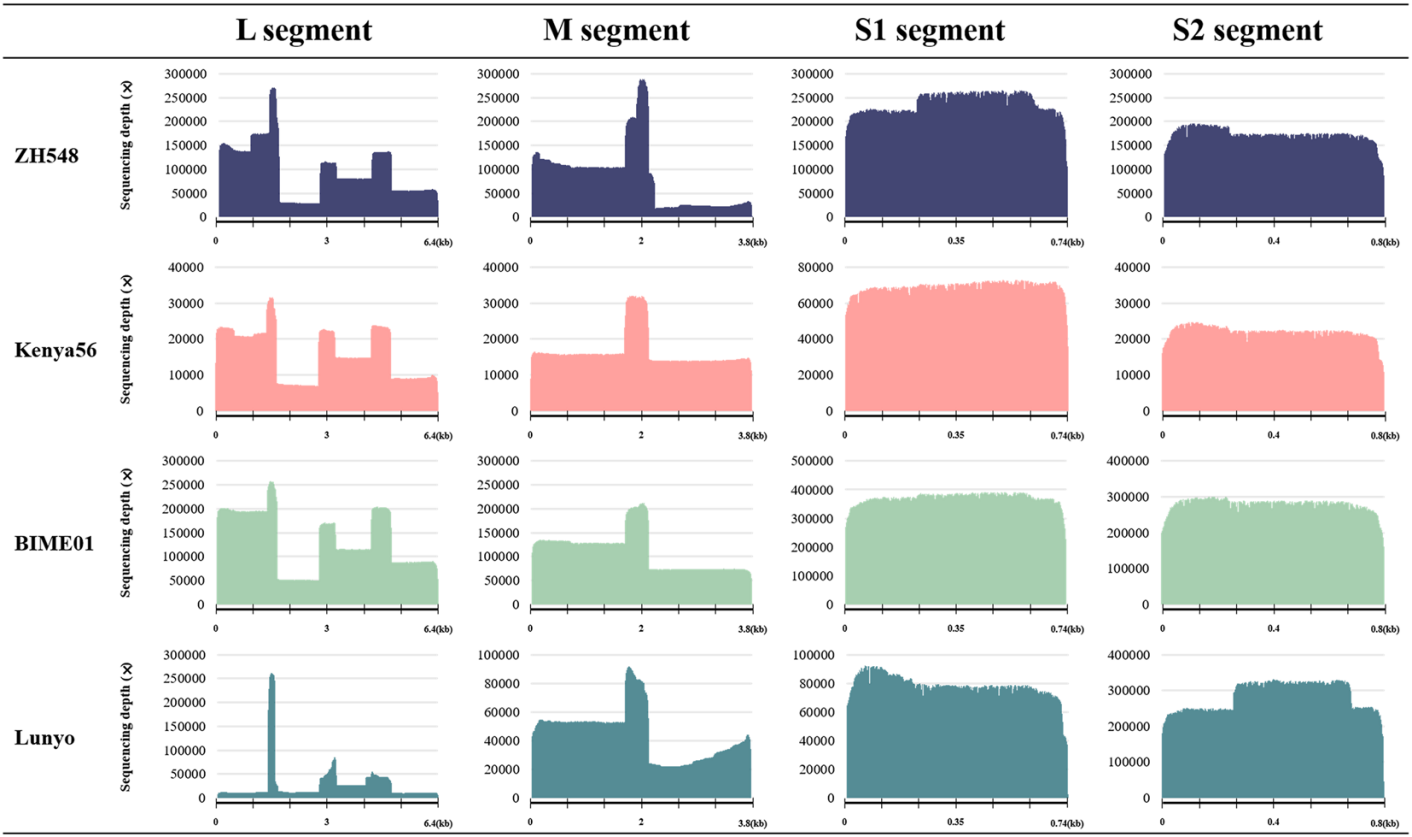
Comparison of primer efficiency among the eight pairs of universal primers measured by sequencing read depth coverage. The MinION sequencing read depth coverage is indicated in the y axis, and the genome region amplified by the eight primer pairs (four pairs in the L segment, two pairs in the M segment, and two pairs in the S segment) is indicated in the x axis. *, S1 product encodes the N gene (738 bp) and S2 product encodes the NSs gene (798 bp).

To compare the amplification efficiency among the eight pairs of universal primers, the read depth coverage of MinION sequences was examined. Overall, the universal primer sets exhibited a high read depth, indicating a high amplification efficiency. Among them, RS01 showed the highest efficiency, while conversely, the performance of RM02 was the lowest (Fig. 4).

### Evaluation of primer sensitivity

The sensitivity of universal primers was assessed by determining the limit of detection. The RNA sample of strain ZH-548 was serially diluted to obtain a viral genome concentration of 10^8^–10^3^ copies/mL. The prepared RNA samples were subjected to PCR and sequencing following the protocol developed in this study. As a result, the entire ZH-548 RVFV genome was successfully assembled to a complete genome at RNA concentrations of 10^4^ copies/mL and above. The sequencing mean depth was 2,288.3–3,015.48X, and sequence similarity to the reference sequence was 99.98–100%. At 10^4^ copies/mL concentration, the lowest sequencing depth was observed from the RL04 primer (248X), and the highest sequencing depth was observed from RM01 primer (12,968X). At RNA concentrations below 10^4^ copies/mL, one or more amplicons among the eight RF primers was not amplified and resulted incomplete genome sequence. Therefore, the detection limit of the developed method for the RVFV samples was determined as 10^4^ copies/mL.

### Clinical specimen applicability

To assess the clinical applicability of the RF primers, a mock clinical specimen was used due to the inaccessibility to human clinical samples. The mock clinical specimen contained a mixture of human blood RNA and RVFV RNA (10^8^ copies/mL). This specimen was subjected to cDNA synthesis, PCR, and sequencing. Even in the mock clinical samples, the entire RVFV genome was successfully amplified and sequenced. The MinION sequencing depth was 23,239.49–1,531,850X and covered the entire genome with 100% sequence similarity.

## Discussion

Virus identification based on whole-genome information allows for precise determination of variants and their lineage. Consequently, whole-genome information is essential for epidemiological investigations and the prevention of disease spread, as it aids in tracing the origin of a specific virus during an outbreak and assessing the potential for dissemination based on known transmission dynamics. This information is vital for controlling the epidemiology and spread of infectious diseases. Traditionally, methods such as sequence-independent single primer amplification (SISPA)^34^, virus discovery cDNA AFLP (VIDISCA)^35^, and rolling circle amplification (RCA)^36^ have been used to obtain the whole genome of viruses. These techniques have the property of amplifying nucleic acids randomly, obviating the need for specific primers. However, such random amplification processes can amplify both the host genome and the target virus genome simultaneously, limiting the efficiency of analyzing the viral genome. With recent advancements in high-throughput sequencing technologies, it has become possible to obtain viral genome sequences directly through shotgun sequencing, bypassing the viral nucleic acid amplification step. However, in cases where the viral concentration is low, such as in clinical samples, the shotgun sequencing approach might not be successful. Due to these reasons, there has been a growing need for universal primers that can effectively amplify very small amounts of viral nucleic acids present in clinical samples, facilitating whole-genome analysis. Universal primers have already found utility in rapidly analyzing genome information and contributing to epidemic management for viruses such as Ebola^37^, influenza^38^, and SARS-CoV-2^39^. The RVFV universal primers developed in this study are also expected to significantly contribute to monitoring and responding to RVF disease in the future.

High-throughput sequencing-based diagnostic methods entail higher costs and time than other viral molecular diagnostic assays, and their sensitivity is relatively lower^40,41^. Accordingly, the whole-genome–based diagnostic method utilizing the universal primers developed in this study exhibits a sensitivity of around ten times lower than the most commonly used RT-PCR method^40^ and demands a longer processing time^41^. The actual experimental cost per sample for genome amplification and sequencing in this study was $300 for MinION and $700 for MiSeq (Supplementary Table 3), being much higher than the cost of few tens of dollars for standard qPCR methods. We are not claiming that the genome analysis method developed in this study can replace standard diagnostic methods. RVF is an extremely dangerous disease, and all suspected cases should be diagnosed accurately through proper standardized testing methods. Considering factors such as sensitivity in clinical samples, cost, and working time, it is appropriate to perform precise diagnosis for patients using traditional standard diagnostic methods. As a secondary step, we propose using the whole-genome analysis method developed in this research for additional mutation analysis.

The RVFV genome encodes virus-derived siRNAs (vsiRNAs), which are evenly distributed throughout the L and M segments of the genome but are concentrated in specific regions of the S segment^42^. Within the S segment, vsiRNAs were primarily clustered in the intergenic region (IGR). Similarly, several bunyaviruses have been found to have dense distributions of vsiRNAs in the IGR, and these are predicted to have a stem and loop (hairpin) structure^42–44^. Such stem and loop structures have been found to reduce the efficiency of polymerases, thereby lowering the efficiency of the overall PCR^44^. Consistently, compared to other amplification products, we obtained relatively lower yields of the S-segment amplicons, which we believe is attributable to the stem and loop structure of the S segment (Supplementary Fig. 4). To overcome this limitation, as an alternative to the RSL primer that covers the entire S segment, we designed two primers (RS01 and RS02) that, with the exception of the central IGR, contained normal coding sequences. Using these primers, we obtained amplification products of the expected sizes for all four strains. RS01 and RS02 were selected as the primers for S-segment amplification.

Unlike other RNA viruses, RVFV lacks well-defined variable regions and is known to have random single-site mutations throughout the genome^14^. Given these characteristics, entire genomes rather than specific genes have been used for phylogenetic analysis. Consequently, the topological resolution tends to be lower for lineages with a small number of reported genomes (lineages B, D, J, M, and O)^30^. Thus, it is necessary to identify a phylogenetic marker with higher resolution, and in this regard, Grobbelaar et al. proposed analysis using the glycoprotein (*Gn*) gene, which has the highest rate of mutation^15,45^. However, discrepancies between the lineages derived from genome phylogenetic analysis and those derived from *Gn* phylogenetic analysis have been reported in some viral strains. For example, while the M and S segments of strain 73HB1230 have been shown to belong to lineage B, the L segment belongs to lineage A^16^. This anomaly is attributable to viral reassortment, which is frequently observed in segmented RNA viruses^45^. Consequently, for accurate phylogenetic analysis of RVFV and identification of mutations, it is necessary to use whole-genome information rather than limited sequences of the *Gn* gene.

To establish universal applicability of the developed protocol, it was necessary to evaluate a broad range of RVFV lineages. Currently, however, there is limited availability of RVFV nucleic acids. To overcome these limitations, we generated synthetic genomes of three genetically different strains using the IVT method. Viral genomic RNA synthesis using IVT is a method that has previously been used for *Ebola Zaire* virus detection and quantification^45^ and for the development of a real-time PCR system for SARS-CoV-2 diagnosis^46^. Thus, it is assumed that our method would be equally applicable in experiments using actual RVFV nucleic acids.

The main limitation of this study is the lack of clinical specimens from RVFV-infected patients. While the primers we developed showed promising results in in vitro experiments, the presence of various inhibitors within actual clinical specimens could introduce false positives. However, since South Korea has never experienced an outbreak of RVF, it is not feasible to import samples related to this virus into the country. Thus, mock RVFV clinical specimens were prepared and assessed instead. Even though RNA extracted from adult male blood, which can act as a significant inhibitor, was included in the mock clinical specimens as non-targeted RNA, the experimental results confirmed successful amplification and sequencing of the entire RVFV genome in the mock clinical samples. In addition, blood viral concentrations of approximately 10^4^–10^8^ copies/mL have been detected within a day after RVFV infection^47^. Thus, it can be assumed that human and animal blood specimens would contain more than the minimum amount (≥ 10^4^ copies/mL) of virus required for the effective use of the developed method. Therefore, it is anticipated that the RVFV universal primer pairs and the experimental method developed in this study will work successfully in real clinical specimens, similar to the way it operated in the mock clinical samples.

## Methods

### Preparation of RVFV nucleic acid

Four representative RVFV strains (ZH548, Kenya 56 (IB8), BIME-01, and Lunyo) were selected and used. ZH548 (lineage A) was chosen because this strain is the most representative experimental strain of RVFV. Kenya 56 (lineage L) and Lunyo (Lineage K) were chosen considering their phylogenetic distance and lineage diversity. BIME-01 was chosen due to its recent transmission from Angola to China in 2016^32^.

The RNA of the Lunyo strain (EVAg 005N-02174, accession no. KU167025, KU167026, and KU167027) was obtained from the Department of Health: Public Health England—Virology & Pathogenesis group (DH) though European Virus Archive Global (EVAg; http://www.european-virus-archive.com/). Lunyo strain RNA was extracted from the supernatants of virus-infected cells and obtained at concentrations of 9.34 × 10^5^ to 3.21 × 10^6^ copies/μL according to EVAg. Genomic DNA of the L, M, and S segments of strains ZH548 (NC_014397, NC_014396, NC_014395), Kenya 56 (DQ375427, DQ380190, DQ380176), and BIME-01(KX609031, KX609032, KX609033) were chemically synthesized by a commercial service (Invitrogen, Waltham, MA, USA) based on the known genome sequence information. The L and S segments of genomic DNA were inserted into a pMA-RQ (AmpR) vector, and the M segment was inserted into a pMA (AmpR) vector (Supplementary Fig. 5A). Recombinant plasmid DNA was used to transform *Escherichia coli* cells using a TOPcloner™ PCR Cloning Kit (Enzynomics, Daejeon, Korea), and the transformed *E. coli* K12 DH10B™ T1R was stored at − 80 °C.

### Preparation of genomic RNAs from synthesized viral genomes

To prepare viral RNAs from the synthesized genome, the recombinant plasmid DNA was linearized using a restriction enzyme at 37 °C for 60 min. The linearized plasmid DNA was used as a template for in vitro transcription (IVT) using T7 RNA polymerase promoter (Supplementary Fig. 5B). Promoter synthesis was performed in a final volume of 25 μL, containing 2 μL of linear plasmid template, 1 μL of each of the forward and the reverse primers (10 μM), 0.5 μL of Ex Taq polymerase (5 U/μL), 2.5 μL of 10X buffer (20 mM), 4 μL of dNTP mix (2.5 mM each), and 14 μL of nuclease-free water. The reaction consisted of initial denaturation at 94 °C for 2 min; followed by 30 cycles of denaturation at 94 °C for 30 s, annealing at 55 °C for 30 s, and extension at 72 °C for 1 min; and a final extension step at 72 °C for 10 min. RVFV genomic RNA was synthesized using the MEGAscript T7 Transcription Kit (Invitrogen) following the manufacturer’s protocol. The reaction mixture was set up in a total volume of 20 μL, comprising 1 μL of linear template DNA (0.1–0.2 μg), 8 μL of NTP solution (75 mM), 2 μL of 10X buffer, 2 μL of enzyme mix, and 7 μL of nuclease-free water. The mixture was then incubated for 2 h. The RNA obtained was purified using Clean & Concentrator™-5 (Zymo Research, Irvine, CA, USA). The quality of RNA used for IVT was evaluated using a BioSpectrometer (Eppendorf, Hamburg, Germany) and a 4150 TapeStation system (Agilent Technologies, Santa Clara, CA, USA). The A260/A280 ratio of the synthesized IVT RNA, determined by BioSpectrometer, was 1.73–2.42. To quantify RNA, we performed droplet digital PCR using a QX200 system (Bio-Rad Laboratories, Hercules, CA, USA) with Supermix for probes (Bio-Rad Laboratories). The ddPCR mixture was prepared according to the manufacturer’s instructions, and amplifications were performed using the following reaction cycle: reverse transcription at 42 °C for 30 min; enzyme activation at 95 °C for 10 min; 70 cycles of denaturation at 95 °C for 30 s and annealing and extension at 60 °C for 150 s; and a final enzyme deactivation step at 98 °C for 10 min. The concentration of the synthesized IVT RNA, determined by ddPCR, was 4.51 × 10^8^ to 6.32 × 10^11^ copies/μL. For the three synthesized viruses (ZH548, Kenya 56, and BIME-01), viral RNA mimics were prepared by mixing the same copy numbers (10^11^ copies/μL) of L, M, and S segment RNAs.

### Designing universal primer candidates

Complete RVFV genome sequences (142 L, 151 M, and 209 S segments) were acquired from the ViPR database. After aligning the base sequences for each segment, primers were designed for the conserved regions. The primer selection criteria were as follows: (i) amplicon size of 0.8–1.7 kb; (ii) melting temperatures of 50–58 °C; (iii) overlap length > 300 bp; (iv) N base ≤ 2; and (v) primer length between 18 and 23 bp.

### Reverse transcription and PCR amplification

The viral genomic RNAs were subjected to a reverse transcription to generate complementary DNA (cDNA) using a LunaScript RT SuperMix Kit (New England Biolabs, Ipswich, MA, USA) following the manufacturer’s protocol. The total volume of the reaction mixture was 20 μL and consisted of 1 μL RNA template, 4 μL of 5X LunaScript RT supermix, and 15 μL of distilled water. The reverse transcription reaction was carried out under the following conditions: primer annealing at 25 °C for 2 min, cDNA synthesis at 55 °C for 10 min, and heat inactivation at 95 °C for 1 min. The synthesized cDNAs were used as templates for PCR without purification.

The cDNAs were used as PCR template for primer screening. PCRs were performed in a final volume of 20 μL, containing 1 μL of first-strand cDNA, 1 μL of each of the forward and the reverse primers (10 pmol), 10 μL of Q5 High-Fidelity 2X Master Mix (New England Biolabs), and 7 μL of nuclease-free water. The amplification cycle consisted of initial denaturation at 98 °C for 30 s; followed by 30 cycles consisting of denaturation at 98 °C for 5 s, annealing at 55 °C for 30 s, and extension at 72 °C for 2 min; and a final extension step at 72 °C for 2 min. Odd/even primer sets were used for multiplex tiling-PCR amplification. The odd set included each odd-region primer (RL01, RL03, RM01, and RS01), and the even set included each even-region primer (RL02, RL04, RM02, and RS02). The amplification products were purified using a QIAquick® PCR Purification Kit (Qiagen, Hilden, Germany) and confirmed via electrophoresis on 1% agarose gels.

### NGS sequencing and assembly

For each sample, purified amplification products were pooled at the same concentration and sequenced using the Illumina MiSeq platform and the ONT MinION Mk1C sequencer. For long-read sequencing, a sequencing library was prepared using an SQK-LSK109 ligation sequencing kit (ONT) and an EXP-NBD104 native barcoding kit (ONT). According to the protocol recommended by ONT, R9.4.1 flow cells (ONT) were used for 1D amplicon ligation sequencing. The Guppy basecaller ver. 6.0.1 + 652ffd1 (ONT) program was used to conduct GPU calling, with a Q-score greater than 8. The quality of the raw data was evaluated using pycoQC (ver. 2.5.2) and NanoPlot (ver. 1.32.1) software^48,49^. The resultant FASTQ file was assembled using BWA-MEM (ver. 0.7.12)^50^. Read alignment coverage and depth coverage were measured using the bedtool program (ver. 2.30.0)^51^, and the errors in mapped results were further corrected using Pilon (ver.1.24)^52^.

A short-read sequencing library was constructed using the TruSeq Nano DNA Kit (Illumina). Following the recommended protocol, 301-bp paired-end sequencing was performed using the Illumina MiSeq platform. Adapter sequences were trimmed from the raw data using Cutadapt ver. 1.11 + 13.g2af9c15, and the overlapping paired-ends were merged and assembled using CLC workbench 9.5.3. (Qiagen), followed by manual correction using the CodonCode Aligner (CodonCode Corporation).

### Phylogenetic analysis

A total of 37 RVF viral genomes across the 15 lineages were used for phylogenetic analysis (Supplementary Table 2). Multiple sequence alignments were performed using the cluster W algorithm in MEGA software (ver. 7.0.26), and phylogenetic trees were constructed using RAxML (version 8.2.10). The inferred trees were summarized using the tree-sumtree program (ver. 4.4.0). Whole-genome phylogenetic analysis was conducted using the concatenated sequences of large (L), medium (M), and small (S) segments.

### Assessment of clinical applicability

There are several reports on the use of mock clinical specimens when human clinical samples are unavailable. Akyurek et al. prepared SARS-CoV-2 mock clinical specimen by mixing IVT synthesized viral RNA (10^8^ copies/μL) and human total RNA (2.5 ng/μL) in a 1:1 volume ratio^53^. Park et al. used RVFV mock clinical specimens by mixing RVFV RNA and human tissue RNA^54^. As we were unable to source human clinical samples of RVFV-infected patients, a mock clinical specimen was prepared to assess the clinical applicability of the developed method. Human adult normal tissue RNA extracted from the blood of a 24-year-old man was obtained from BioChain (Newark, CA, USA). The mock clinical specimen contained 25 μL of human adult normal tissue RNA (200 ng/μL), 25 μL of RVFV IVT RNA, and 200 μL RNA storage solution (Invitrogen). The final concentration of viral genome in this mock clinical specimen was adjusted to 10^8^ copies/mL. The cDNA synthesis, PCR amplification, MinION sequencing, and assembly were performed as described above.

### Supplementary Information

Supplementary Tables.

Supplementary Figures.

## Data Availability

The genome sequences generated during the current study are available from the NCBI under the accession numbers OQ440142—OQ440157, https://www.ncbi.nlm.nih.gov/nuccore/OQ440142—https://www.ncbi.nlm.nih.gov/nuccore/OQ440157.
