# Supplementary material for: Universal primers for rift valley fever virus whole-genome sequencing
Source: Sci Rep. 2023 Oct 31;13:18688. doi: 10.1038/s41598-023-45848-z (PMC10618441; doi:10.1038/s41598-023-45848-z)
Supplement: Supplementary file 1 — Supplementary Tables. [file 41598_2023_45848_MOESM1_ESM.docx]

Supplementary Material Table

**Supplementary Table 1. Comparison of the whole-genome sequences of four viral strains determined by MiSeq sequencing and *de novo* assembly.**

| **Strains** | **% Genome coverage^*^**  **(L / M / S1 / S2)** | **% Similarity^#^** |
| --- | --- | --- |
| ZH-548 | 99.9844 / - / 100 / 100 | 100 / - / 100 / 100 |
| Kenya 56 (IB8) | 98.8446 / 99.5367 / 100 / 100 | 100 / 100 / 100 / 100 |
| BIME-01 | 100 / 99.9567 / 100 / 100 | 100 / 100 / 100 / 100 |
| Lunyo | 100 / 99.3565 / - / 100 | 99.9688 / 98.4556 / - / 99.8645 |

^*^ Genome coverage is the percentage area covered by a reference sequence (consensus sequence; L, 6,404 bp; M, 3,867 bp; S1, 738 bp; S2, 798 bp) by the assembled contigs.

^#^Similarity means the proportion of matched nucleotides in the assembled contigs compared to a reference.

**Supplementary Table 2. List of RVFV strains included in the phylogenetic analyses.** The complete genome sequences of RVFV were obtained from the NCBI database.

| **Strains** | **Country** | **Source** | **Lineage** | **NCBI Accession number (L/M/S)** |
| --- | --- | --- | --- | --- |
| 2250/74 | Zimbabwe | Unknown | A | DQ375413 / DQ380209 / DQ380143 |
| MgH824 | Madagascar | Human | A | DQ375414 / DQ380210 / DQ380144 |
| ZH-501 | Egypt | Unknown | A | DQ375406 / DQ380200 / DQ380149 |
| ZH-548 | Egypt | Human | A | NC014397 / NC014396 / NC014395 |
| ZM-657 | Egypt | Mosquito | A | DQ375409 / DQ380204 / DQ380146 |
| ZS-6365 | Egypt | Unknown | A | DQ375410 / DQ380205 / DQ380145 |
| ZH-1776 | Egypt | Unknown | A | DQ375411 / DQ380203 / DQ380153 |
| Kenya 83 (21445) | Kenya | Mosquito | B | DQ375402 / DQ380198 / DQ380171 |
| Kenya 9800523 | Kenya | Unknown | C | DQ375400 / DQ380196 / DQ380169 |
| Saudi 2000-10911 | Saudi Arabia | Human | C | DQ375401/ DQ380197 / DQ380170 |
| SA01-1322 | Saudi Arabia | Unknown | C | KX096941 / KX096942 / KX096943 |
| Sudan 2V-2007 | Sudan | Human | C | JQ820483 / JQ820490 / JQ820472 |
| TAN/Tan-001/07 | Tanzania | Human | C | HM586959 / HM586970 / HM586981 |
| Sudan 85-2010 | Sudan | Human | C | JQ820485 / JQ820488 / JQ820476 |
| 2007000234 | Kenya | Human | C | JF326186 / JF326191 / JF326198 |
| 200803162 | Madagascar | Human | C | JF311368 / JF311377 / JF311386 |
| Kenya-128b-15 | Kenya | Mosquito | C | KX096938 / KX096939 / KX096940 |
| 73HB1230 | Central African Republic | Unknown | D | DQ375425 / DQ380221 / DQ380172 |
| 73HB1449 | Central African Republic | Human | E | DQ375416 / DQ380211 / DQ380162 |
| Hv-B375 | Central African Republic | Human | G | DQ375422 / DQ380218 / DQ380161 |
| ANK-6087 | Guinea | Bat | G | DQ375421 / DQ380216 / DQ380166 |
| ANK-3837 | Guinea | Bat | G | DQ375420 / DQ380215 / DQ380165 |
| ArB1986 | Central African Republic | Mosquito | G | KJ782457 / KJ782456 / KJ782455 |
| 2269/74 | Zimbabwe | Unknown | J | DQ375434 / DQ380222 / DQ380173 |
| Kenya 57 (Rintoul) | Kenya | Sheep | K | DQ375431 / DQ380192 / DQ380155 |
| Smithburn | Uganda | Unknown | K | DQ375430 / DQ380193 / DQ380157 |
| Entebbe | Uganda | Mosquito | K | DQ375429 / DQ380191 / DQ380156 |
| BIME-01 | China | Human | K | KX609031 / KX609032 / KX609033 |
| Kakamas | South Africa | Sheep | K | JQ068144 / JQ068143 / JQ068142 |
| Kenya 56 (IB8) | Kenya | Cattle | L | DQ375427 / DQ380190 / DQ380176 |
| 763/70 | Zimbabwe | Cattle | L | DQ375426 / DQ380188 / DQ380174 |
| SA-75 | South Africa | Unknown | L | DQ375428 / DQ380189 / DQ380175 |
| Lunyo | Uganda | Unknown | M | KU167027 / KU167026 / KU167025 |
| ARD-38388 | Burkina Faso | Unknown | N | DQ375399 / DQ380187 / DQ380181 |
| OS-1 | Mauritania | Human | N | DQ375398 / DQ380186 / DQ380180 |
| OS-3 | Mauritania | Human | N | DQ375396 / DQ380184 / DQ380178 |
| SA-51 (Van Wyck) | South Africa | Unknown | O | DQ375433 / DQ380195 / DQ380158 |

**Supplementary Table 3. Experimental cost required per sample for genome amplification and sequencing in this study.** One flow cell run of MinION sequencer performed with six samples and one MiSeq run was performed with three samples. Accordingly, the cost for MinION and MiSeq diagnosis was $300 and $700 per sample, respectively.

| **Component** | **MinION**  **(6 samples)** | **MiSeq**  **(3 samples)** | **Listed price** | **Reaction/pkg** |
| --- | --- | --- | --- | --- |
| NEB Q5 high fedelity DNA polymerase | $ 8.56 | $4.28 | $ 142.71 | 100 |
| dNTPs (2.5mM) | $ 1.57 | $0.79 | $ 32.81 | 125 |
| QIAquick PCR purification kit | $ 19.43 | $7.28 | $ 121.40 | 50 |
| Sequencing kit (LSK-SQT 109) | $ 136.13 |  | $ 816.88 | 36 |
| Native barcoding kit 1-12 (EXP-NBD 104) | $ 32.8 |  | $ 393.68 | 72 |
| Flow cell (FLO-MIN 106D) | $ 1,312.26 |  | $ 1,312.26 | 6 |
| Agencourt AMPure XP | $ 43.84 |  | $ 241.16 | 33 |
| Blunt/TA ligase master mix | $ 15.35 |  | $ 127.96 | 50 |
| NEBNext FFPE DNA repair mix | $ 57.00 |  | $ 228.04 | 24 |
| NEBNext Ultra II end repair dA-tailing module | $ 86.53 |  | $ 346.16 | 24 |
| NEBNext Qick ligation module | $ 129.67 |  | $ 432.29 | 20 |
| TruSeq Nano DNA HT LPK (96 spl) |  | $ 98.50 | $ 3,151.99 | 96 |
| TruSeq DNA UD Index v2 |  | $ 22.9 | $ 731.12 | 96 |
| PhiX Control v3 |  | $ 6.17 | $ 205.84 | 100 |
| MiSeq® Reagent Kit v3 (600 cycle) |  | $ 1,940.12 | $ 1,940.12 | 3 |
| **Total cost** | **$ 1,843.14** | **$ 2,085.10** |  |  |
| **Cost per sample** | **$ 307.19** | **$ 695.03** |  |  |
