# Supplementary material for: Universal primers for rift valley fever virus whole-genome sequencing
Source: Sci Rep. 2023 Oct 31;13:18688. doi: 10.1038/s41598-023-45848-z (PMC10618441; doi:10.1038/s41598-023-45848-z)

Supplementary Figure 1. The tiling PCR products of RVFV universal primers.

Agarose gel electrophoresis image of PCR amplicons from the tiling PCR of ZH548, Kenya 56 (IB8), BIME-01, and Lunyo.

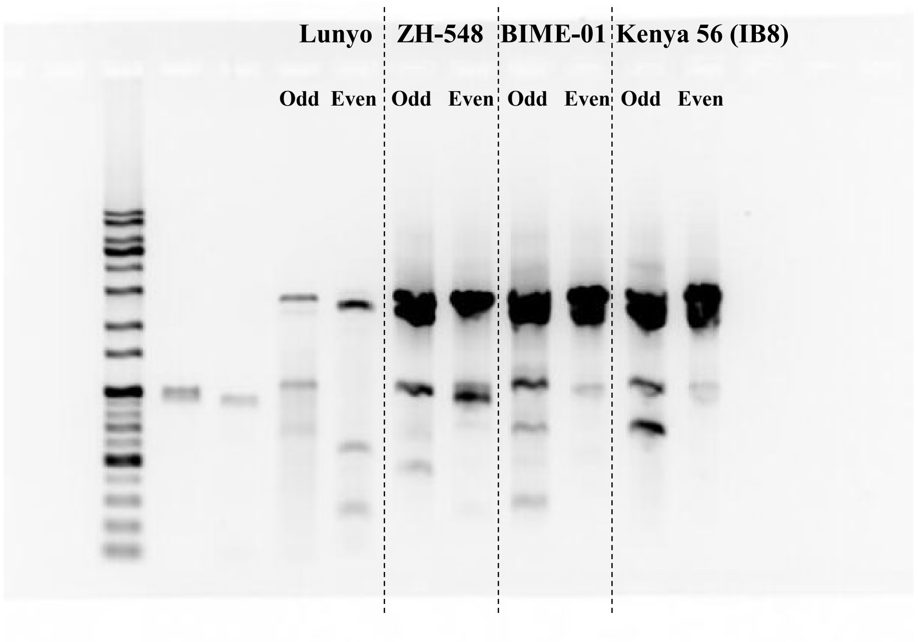

Supplementary Figure 2. Phylogenetic tree based on the Gn gene sequence of RVFV.

Maximum likelihood analysis was performed using RAxML version 8.2.10 and evaluated using 100,000 bootstrap replicates. Lineage names followed the nomenclature of Grobbelaar et al.[11]

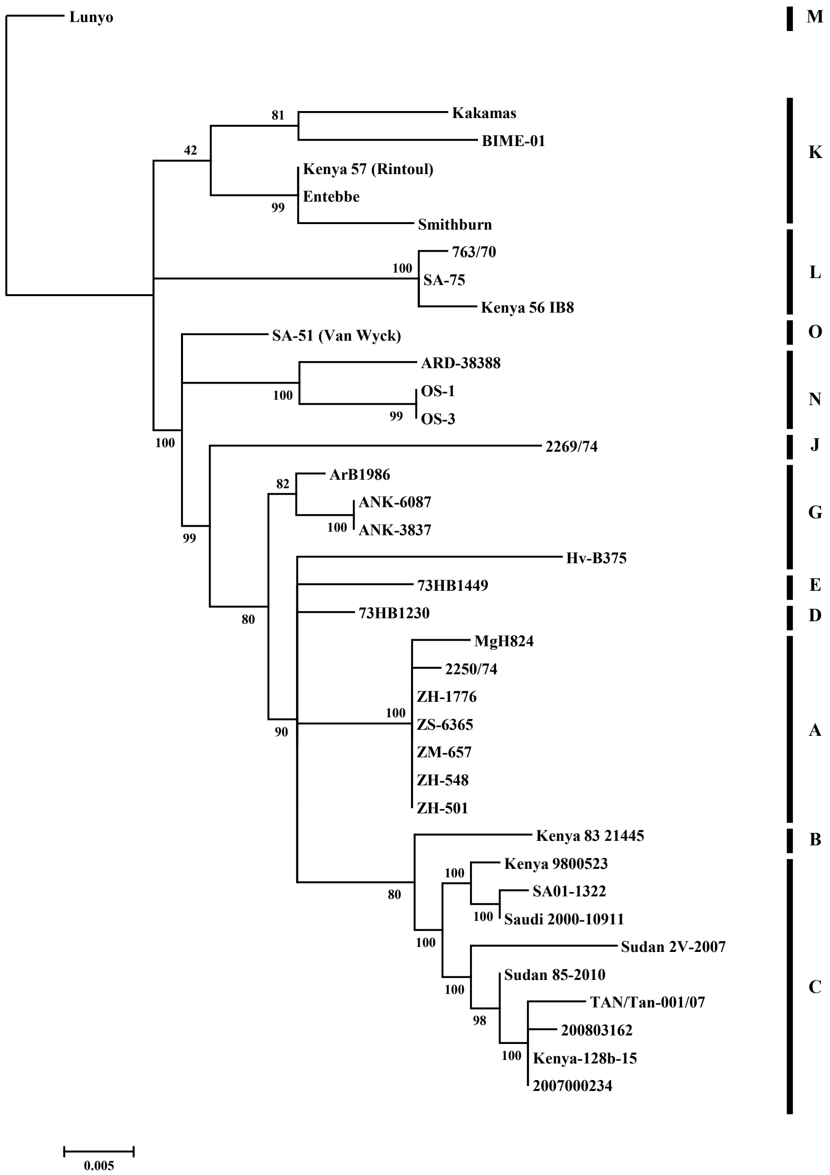

Supplementary Figure 3. Primer mismatches observed in the 12 lineages of RVFV.

The designed primers were aligned to the representative RVFV genome. The observed mismatches between individual primers and genomic sequences are shown.

	Lineage	RL01		RL02		RL03		RL04		RM01		RM02		RS01		RS02		Total
		F	R	F	R	F	R	F	R	F	R	F	R	F	R	F	R	
1) 2250/74|DQ380209|Unknown|Rift-Valley-fever-phlebovirus	A	0	0	0	0	0	0	0	0	0	0	0	0	0	0	0	0	0
2) MgH824|DQ380210|Human|Rift-Valley-fever-phlebovirus	A	0	0	0	0	0	0	0	0	0	0	0	0	0	0	0	0	0
3) ZH-501|DQ380200|Unknown|Rift-Valley-fever-phlebovirus	A	0	0	0	0	0	0	0	0	0	0	0	0	0	0	0	0	0
4) ZH-548|NC-014396|Human|Rift-Valley-fever-phlebovirus	A	0	0	0	0	0	0	0	0	0	0	0	0	0	0	0	0	0
5) ZM-657|DQ380204|Mosquito|Rift-Valley-fever-phlebovirus	A	0	0	0	0	0	0	0	0	0	0	0	0	0	1	0	0	1
6) ZS-6365|DQ380205|Unknown|Rift-Valley-fever-phlebovirus	A	0	0	0	0	0	0	0	0	0	0	0	0	0	0	0	0	0
7) ZH-1776|DQ380203|Unknown|Rift-Valley-fever-phlebovirus	A	0	0	0	0	0	0	0	0	0	0	0	0	0	0	0	0	0
8) Kenya 83 21445|DQ380198|Mosquito|Rift-Valley-fever-phlebovirus	B	0	0	0	0	0	0	0	0	0	0	0	0	0	0	0	0	0
9) Kenya 9800523|DQ380196|Unknown|Rift-Valley-fever-phlebovirus	C	0	1	0	0	0	0	0	0	0	0	0	0	0	0	0	0	1
10) Saudi 2000-10911|DQ380197|Human|Rift-Valley-fever-phlebovirus	C	0	1	0	0	0	0	0	0	0	0	0	0	0	0	0	0	1
11) SA01-1322|KX096942|Unknown|Rift-Valley-fever-phlebovirus	C	0	1	0	0	0	0	0	0	0	0	0	0	0	0	0	0	1
12) Sudan 2V-2007|JQ820490|Human|Rift-Valley-fever-phlebovirus	C	0	0	0	0	0	0	0	0	0	0	0	0	0	0	1	0	0
13) TAN/Tan-001/07|HM586970|Human|Rift-Valley-fever-phlebovirus	C	1	0	0	0	0	0	0	0	0	0	0	0	3	0	1	0	4
14) Sudan 85-2010|JQ820488|Human|Rift-Valley-fever-phlebovirus	C	0	1	0	0	0	0	0	0	0	0	0	0	0	0	0	0	1
15) 2007000234|JF326191|Human|Rift-Valley-fever-phlebovirus	C	0	0	0	0	0	0	0	0	0	0	0	0	0	0	0	0	0
16) 200803162|JF311377|Human|Rift-Valley-fever-phlebovirus	C	0	0	0	0	0	0	0	0	0	0	0	0	0	0	0	0	0
17) Kenya-128b-15|KX096939|Mosquito|Rift-Valley-fever-phlebovirus	C	0	0	0	0	0	0	0	0	0	0	0	0	0	0	0	0	0
18) 73HB1230|DQ380221|Unknown|Rift-Valley-fever-phlebovirus	D	0	0	0	0	0	0	0	0	0	0	0	0	0	0	0	0	0
19) 73HB1449|DQ380211|Human|Rift-Valley-fever-phlebovirus	E	0	0	0	0	0	0	0	0	0	0	0	0	0	1	0	0	1
20) Hv-B375|DQ380218|Human|Rift-Valley-fever-phlebovirus	G	0	0	0	0	0	0	0	0	0	0	0	0	0	1	0	0	1
21) ANK-6087|DQ380216|Bat|Rift-Valley-fever-phlebovirus	G	0	0	0	0	0	0	0	0	0	0	0	0	0	1	0	0	1
22) ANK-3837|DQ380215|Bat|Rift-Valley-fever-phlebovirus	G	0	0	0	0	0	0	0	0	0	0	0	0	0	1	0	0	1
23) ArB1986|KJ782456|Mosquito|Rift-Valley-fever-phlebovirus	G	0	0	0	0	0	0	0	0	0	0	0	0	0	1	0	0	1
24) 2269/74|DQ380222|Unknown|Rift-Valley-fever-phlebovirus	J	0	0	0	0	0	1	1	0	0	1	1	0	0	0	0	0	4
25) Kenya 57 Rintoul|DQ380192|Sheep|Rift-Valley-fever-phlebovirus	K	0	0	0	0	0	0	0	0	0	0	0	0	0	0	0	0	0
26) Smithburn|DQ380193|Unknown|Rift-Valley-fever-phlebovirus	K	0	0	0	0	0	0	0	0	0	0	0	0	0	0	1	0	0
27) Entebbe|DQ380191|Mosquito|Rift-Valley-fever-phlebovirus	K	0	0	0	0	0	1	0	0	0	0	0	0	0	0	0	0	1
28) BIME-01|KX609032|Human|Rift-Valley-fever-phlebovirus	K	0	0	0	0	0	0	0	0	0	0	0	0	0	0	0	0	0
29) Kakamas|JQ068143|Sheep|Rift-Valley-fever-phlebovirus	K	0	0	0	0	0	0	0	0	0	1	0	0	0	0	1	0	1
30) Kenya 56 IB8|DQ380190|Cattle|Rift-Valley-fever-phlebovirus	L	0	0	0	0	0	0	0	0	0	0	0	0	0	1	0	0	1
31) 763/70|DQ380188|Cattle|Rift-Valley-fever-phlebovirus	L	0	0	0	0	0	1	0	0	0	1	0	0	0	1	0	0	3
32) SA-75|DQ380189|Unknown|Rift-Valley-fever-phlebovirus	L	0	0	0	0	0	0	0	0	0	0	0	0	0	1	0	0	1
33) Lunyo|KU167026|Unknown|Rift-Valley-fever-phlebovirus	M	0	0	0	0	0	0	0	0	0	0	0	0	0	0	1	0	0
34) ARD-38388|DQ380187|Unknown|Rift-Valley-fever-phlebovirus	N	0	0	0	0	0	0	0	0	0	0	0	1	0	1	2	0	2
35) OS-1|DQ380186|Human|Rift-Valley-fever-phlebovirus	N	0	0	0	0	0	0	0	0	0	0	0	0	0	0	1	0	0
36) OS-3|DQ380184|Human|Rift-Valley-fever-phlebovirus	N	0	0	0	0	0	0	0	0	0	0	0	0	0	0	1	0	0
37) SA-51 Van Wyck|DQ380195|Unknown|Rift-Valley-fever-phlebovirus	O	0	0	0	0	0	0	1	0	0	0	0	2	0	0	0	0	3

Supplementary Figure 4. PCR amplification efficiencies of the designed primers measured by amplicon concentration.
The DNA concentration in the PCR product varied depending on the primers, showing an amplification efficiency bias across the RVFV genomic region.
* RSL = Rift Valley fever virus S segment long primer.

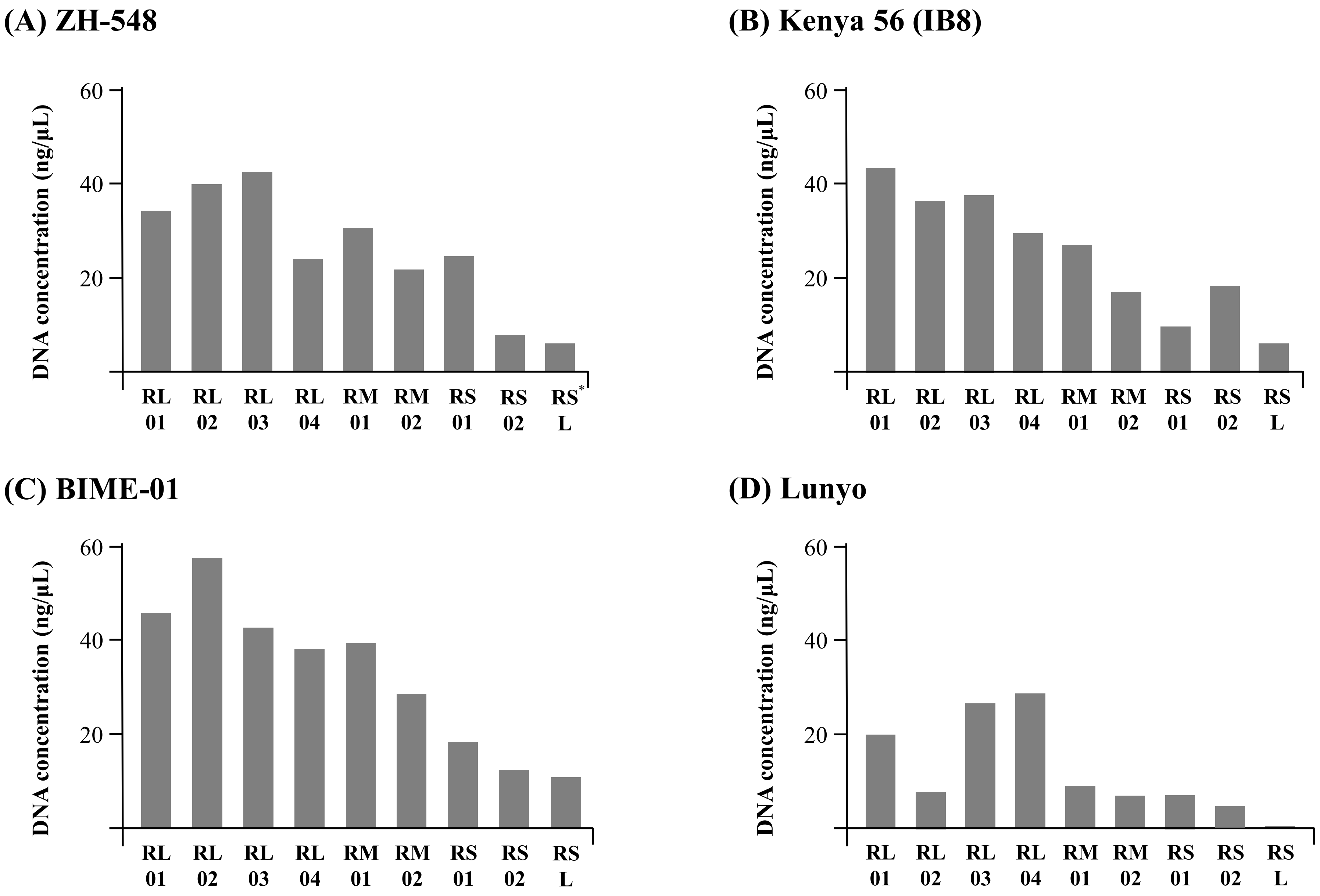

Supplementary Figure 5. The recombinant plasmid DNA containing the chemically synthesized RVFV genome segments used for in vitro transcription.

(a) Genomic DNA of the L, M, and S segments of strains ZH548, Kenya 56, and BIME-01 were chemically synthesized and prepared as recombinant plasmids. (b) The recombinant plasmid DNA was linearized and then in vitro transcribed using T7 RNA polymerase promoter to serve as the template for RVFV genomic RNA synthesis.

(A)

RVFV	ZH-548, Kenya 56 (IB8), BIME-01		
Segment	L	M	S
Size (bp)	6,416	3,897	1,703
Vector	pMA	pMA-RQ	pMA
Recombinant plasmid	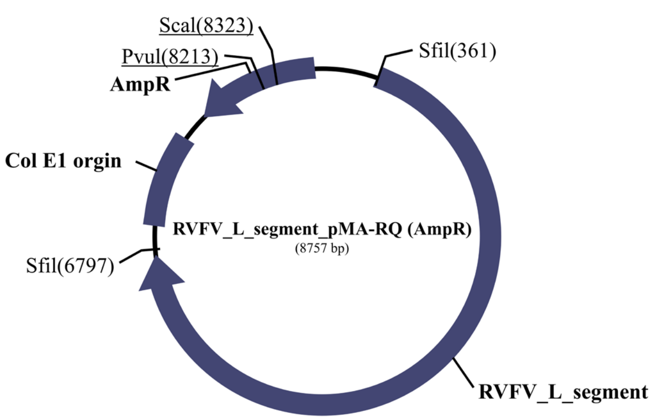	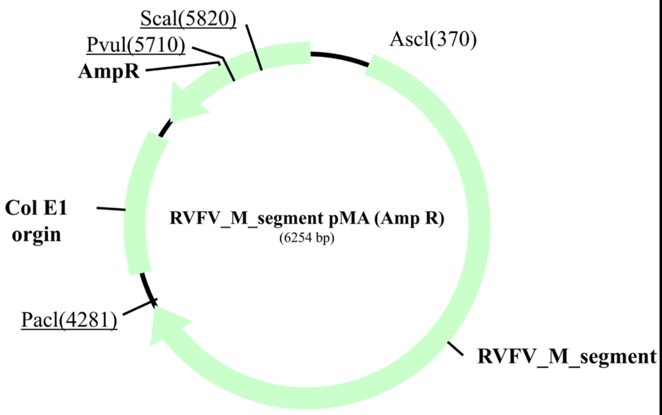	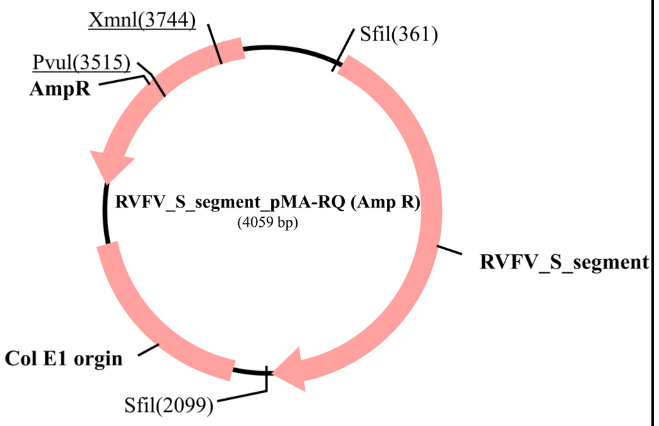

(B)

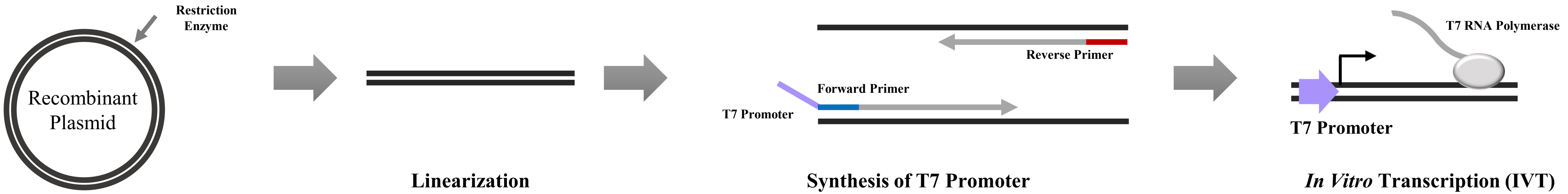

Supplement: Supplementary file 2 — Supplementary Figures. [file 41598_2023_45848_MOESM2_ESM.pdf]
